# Mitochondrial Bioenergetics and Dynamics in Secretion Processes

**DOI:** 10.3389/fendo.2020.00319

**Published:** 2020-05-22

**Authors:** Jennyfer Martínez, Inés Marmisolle, Doménica Tarallo, Celia Quijano

**Affiliations:** Departamento de Bioquímica, Centro de Investigaciones Biomédicas (CEINBIO), Facultad de Medicina, Universidad de la República, Montevideo, Uruguay

**Keywords:** mitochondria, secretion, bioenergetics, ATP, calcium, dynamics, endoplasmic reticulum, exocytosis

## Abstract

Secretion is an energy consuming process that plays a relevant role in cell communication and adaptation to the environment. Among others, endocrine cells producing hormones, immune cells producing cytokines or antibodies, neurons releasing neurotransmitters at synapsis, and more recently acknowledged, senescent cells synthesizing and secreting multiple cytokines, growth factors and proteases, require energy to successfully accomplish the different stages of the secretion process. Calcium ions (Ca^2+^) act as second messengers regulating secretion in many of these cases. In this setting, mitochondria appear as key players providing ATP by oxidative phosphorylation, buffering Ca^2+^ concentrations and acting as structural platforms. These tasks also require the concerted actions of the mitochondrial dynamics machinery. These proteins mediate mitochondrial fusion and fission, and are also required for transport and tethering of mitochondria to cellular organelles where the different steps of the secretion process take place. Herein we present a brief overview of mitochondrial energy metabolism, mitochondrial dynamics, and the different steps of the secretion processes, along with evidence of the interaction between these pathways. We also analyze the role of mitochondria in secretion by different cell types in physiological and pathological settings.

## Introduction

About 20% of the proteins synthesized by eukaryotic cells are secreted to the extracellular space either as soluble or membrane bound proteins (1). The secretory pathway in eukaryotic cells is responsible for biogenesis and proper distribution of a wide range of extracellular proteins, as well as complex carbohydrates and lipids. This pathway is highly dynamic and responsive to specific cellular demands and stimuli (2). Eukaryotic cells also secrete many amino acids and amino acid derivatives, such as neurotransmitters, that play key roles in intercellular communication (3).

As many other complex cellular processes secretion of proteins consumes energy, therefore requires the support of functional mitochondria. In the conventional pathway proteins are transported into the endoplasmic reticulum (ER), and folding and quality control of proteins in the ER (4–8) consumes ATP. Likewise the assembly, transport and fusion of the vesicles carrying proteins from the ER to the Golgi and to the plasma membrane requires the hydrolysis of ATP, as well as GTP (2, 9, 10). Besides, several steps in the secretion process depend on calcium ion (Ca^2+^) as a cofactor or signaling molecule (11, 12). Calcium pumps are required to maintain the appropriate concentrations of Ca^2+^ inside the ER and near the sites of exocytosis, and mitochondria play a relevant role providing energy to transport Ca^2+^ against its concentration gradient (13, 14). Mitochondria also modulate Ca^2+^ concentrations in the cytosol, sequestering the ion in the mitochondrial matrix (14, 15). Secretion of proteins by unconventional pathways also depends on mitochondria, not only as a source of ATP, but also of activation signals such as mitochondrial reactive oxygen species (ROS) and oxidized mtDNA (16, 17), as well as a structural platform for the assembly of inflammasomes (17, 18).

Work by others and us shows that impairment of mitochondrial catabolism and dynamics affects the secretion processes (6, 7, 19–26) and can underlie pathology in endocrine and neurodegenerative diseases (27–30). Relevant roles for mitochondria in protein secretion by immune and senescent cells have been recently described as well (19–21, 24–26, 31), underscoring the relevance of these interactions. This prompted us to explore the existing literature on mitochondrial interactions with the secretion machinery.

**Figure 1:**
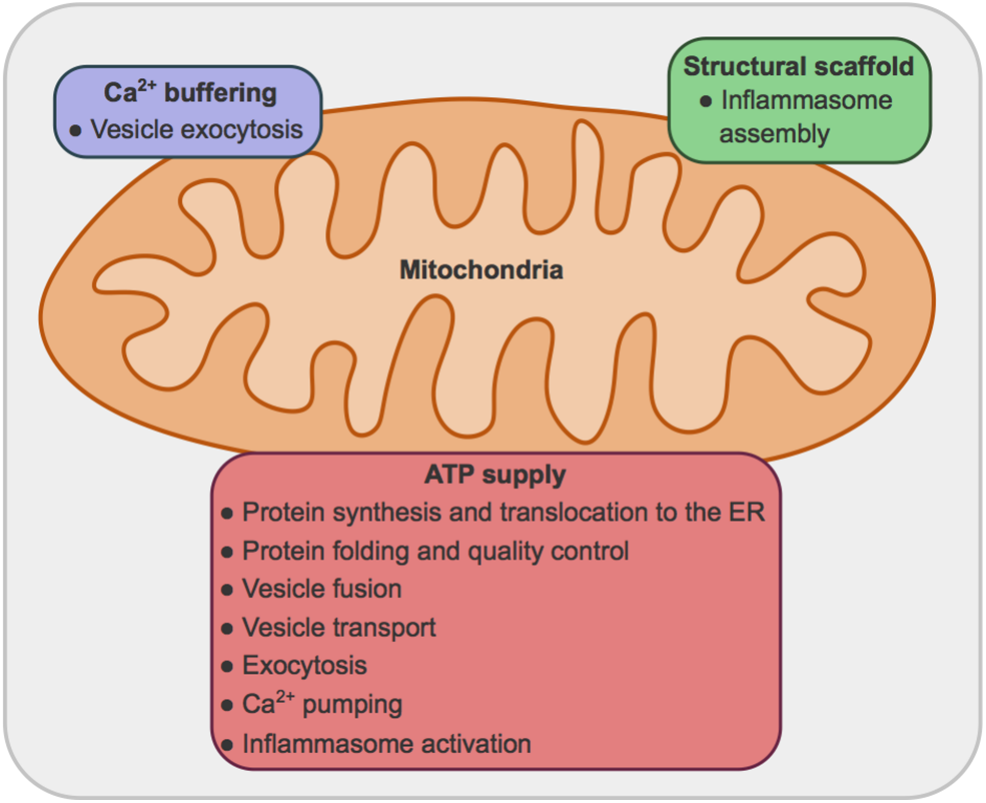
Main roles for mitochondria in secretory processes. (1) Mitochondria provide ATP, obtained by oxidative phosphorylation, for: protein synthesis, translocation to the ER, folding and quality control, vesicle transport, vesicle fusion and exocytosis, Ca^2+^ pumping across plasma and ER membranes; and inflammasome activation. (2) Mitochondria can uptake Ca^2+^, modulating Ca^2+^ concentration and therefore vesicle exocytosis. (3) Mitochondria provide a structural scaffold for the assembly of the NLRP3 inflammasome.

While extensive literature can be found regarding both secretion and mitochondrial bioenergetics and dynamics, the connection between these processes and the organelles involved are still largely unexplored. In this manuscript secretion pathways, mitochondrial metabolism and dynamics *per se* have not been extensively reviewed. We have relied on reviews by others to present an overview, and centered our efforts in exploring the requirement for mitochondrial ATP and fusion and fission proteins to sustain secretion in metazoans, excluding the events linked to ER stress. We expand on three main roles of mitochondria: (1) providing ATP for multiple steps of the secretion process; (2) buffering Ca^2+^ concentrations; (3) providing signals and structural scaffold for the activation of the inflammasome (Figure 1).

## Overview of the Secretion Pathways and Their Energy Demands

**Figure 2:**
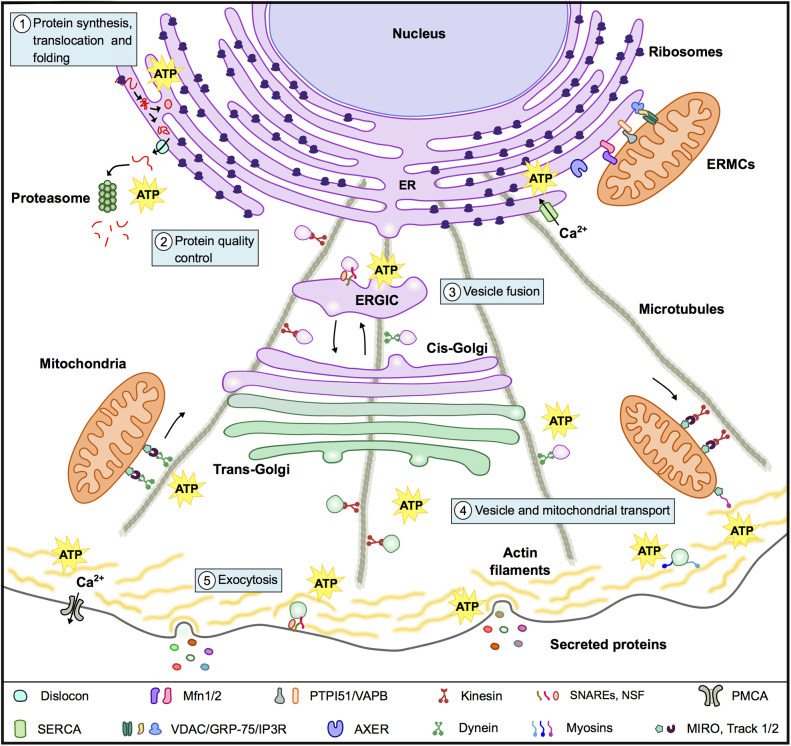
Mitochondria and the conventional secretion machinery. To support the energy requirements of the secretion process mitochondria interact with organelles and components of the cytoskeleton and supply ATP for: (1) Protein synthesis, translocation to the ER and folding. (2) Protein quality control in particular for the energy consuming ERAD. (3) Vesicle fusion with target membranes in the ER, ERGIC, Golgi, and plasma membrane. (4) Vesicle and mitochondrial transport along microtubules and actin filaments. (5) Exocytosis. In the panel below the figure are a series of molecules and complexes that play relevant roles in these events.

Secreted proteins can reach the extracellular media through the conventional (classic) pathway or through unconventional pathways (32). In the conventional pathway proteins are transported into the ER, either cotranslationally or postranslationally; then travel from the ER to the Golgi complex, from which they then migrate to the trans-Golgi network and finally to the plasma membrane (33, 34). Transport from one compartment to another takes place by sequential budding and fusion of vesicles (35) and the microtubule and actin cytoskeleton plays a relevant role in vesicle transport (33, 36) (Figure 2). Unconventional secretion pathways, on the other hand, include proteins that do not present a leader sequence and proteins that by-pass the Golgi apparatus while traveling to the plasma membrane (32).

### Conventional Pathway for Protein Secretion

Most of the secreted proteins in the conventional pathway are translocated to the rough ER during translation by the ribosome (37). Proteins are targeted to the ER by a hydrophobic signal peptide located in the N-terminus, which is later cleaved by a signal peptidase present in the ER lumen. The signal peptide is recognized by the signal recognition particle (SRP) that then binds to the SRP receptor (SR) (33, 38). Both SRP and SR are GTPases and the docking and release of SRP at the ER membrane requires GTP (37, 39). Docking is followed by the translocation of the nascent protein to the ER lumen through the translocon, a pore that in mammals is composed of Sec61 proteins, and associated proteins including BiP, Sec63, Sec62, translocating-chain-associated protein (TRAM), translocon-associated protein (TRAP), and ribosome-associated membrane protein (RAMP) (37, 40, 41). BiP plays a dual role during protein translocation, it seals de pore and provides the driving force to transfer of the nascent protein into the ER (4, 40). This protein is an ATP-dependent chaperone that belongs to the heat shock protein 70 (Hsp70) family. During protein translocation BiP interacts with co-chaperones Sec63 and RAMP, these ER-resident J-domain proteins (ERdjs) stimulate BiP binding to the nascent protein and ribosomes as well as ATP hydrolysis (4, 41). BiP also interacts with the nucleotide exchange factors GRP170 and Sil1 that promote the exchange of ADP for ATP (41, 42). Though ATP hydrolysis by BiP is the main driving force for translocation, GTP hydrolysis during the elongation stage of protein synthesis may also contribute to the process (4, 40) (Table 1 and Figure 2).

**Table 1:** The conventional pathway for protein secretion and its energy requirements.

**Process**	**Associated proteins**	**References**
Protein synthesis	**Aminoacyl-tRNA synthetases**, initiation factors, elongation factors, termination factors, ribosomes	(43)
Protein translocation	SRP, SRP receptor, SEC61, SEC62, SEC63, **BiP**, GRP170, SIL1, TRAM, TRAP	(4, 40)
Protein folding	OGT, exoglucosidases I and II, calnexin, calreticulin, GT, PDIA3, **BiP, GRP-94**	(41, 44)
Protein quality control	ER α1,2-mannosidase, EDEMs 1/2/3, **BiP**, PDIs, OS-9, XTP-3B, SEL1L, **ubiquitin activating enzyme (E1)**, ubiquitin conjugating enzyme (E2), ubiquitin ligase (E3, HRD1), SEC61, derlins, **ATPase VCP/p97, 26S proteasome**	(41, 45, 46)
COPII and COPIvesicle assembly	SAR1, ARF, SEC12, SEC16, SEC23, SEC24, SEC13, SEC31, COPI subunits, GEFs, GAPs	(47, 48)
Vesicle fusion	Receptors, Rab GTPases, Rab effectors SNARE proteins, SM proteins, SNAP, **NSF**	(2)
Vesicle transport	**Kinesin, dynein, myosins**	(9, 49)
Exocytosis	Rab GTPases, Rab effectors SNARE proteins, SM proteins, SNAP, **NSF, actin, myosins**	(50, 51)

*Relevant molecules involved in the different steps of the conventional secretion pathway are listed. Proteins that catalyze ATP hydrolysis are highlighted in bold letters*.

In the ER, the oligosaccharyl transferase complex (OGT) catalyzes the transfer of the oligosaccharide (Glc_3_Man_9_GlcNAc_2_) from dolichol phosphate to an asparagine residue in the protein (52, 53). Exoglucosidases I and II then catalyze the removal of two terminal glucose moieties producing a monoglucosylated structure (Glc_1_Man_9_GlcNAc_2_) that is recognized and bound by lectin chaperones calnexin and calreticulin. These chaperones, in collaboration with BiP, GRP-94 and protein disulfide isomerase A3 (PDIA3, Erp57), help the protein acquire its native conformation (8, 38). Removal of the remaining glucose moiety by exoglucosidase II prevents further binding of the glycoprotein to calnexin and calreticulin, allowing it exit to the ER. However, if the native structure is not achieved the protein undergoes reglucosylation catalyzed by UDP-glucose:glycoprotein glucosyltransferase (GT), an enzyme that preferentially acts on incompletely folded glycoproteins. Thereby favoring a new cycle of interactions with calnexin and calreticulin and the other components of the folding machinery (8, 54, 55). Protein folding requires ATP, since chaperones BiP and GRP-94 hydrolyze ATP during their catalytic cycles (41, 44). Besides, calnexin and calreticulin bind ATP though they do not hydrolize it (56). Calnexin and calreticulin also bind Ca^2+^, therefore Ca^2+^ homeostasis must be maintained in the ER lumen (56). The sarcoplasmic/endoplasmic reticulum calcium ATPase (SERCA) is responsible of coupling ATP hydrolysis to Ca^2+^ transport from the cytosol to the ER (4, 57) (Figure 2).

In the ER proteins undergo quality control pathways for the detection, remotion, and degradation of misfolded proteins that did not attain their native structures. Degradation by the proteasome takes place in the cytosol in a pathway known as ER-associated degradation (ERAD) (8, 45). The first step in this pathway is the recognition of the misfolded protein and several luminal and cytoplamatic factors are involved in this process (46). Among them we find ER mannosidases (ER α1,2-mannosidase and EDEMs, 1, 2, and 3) that trim the mannose residues from glycoproteins, preventing glucosylation by GT and therefore withdrawing the protein from the quality control cycle (8, 58). Mannose trimmed glycoproteins bind OS-9 and XTP-3B and associate with SEL1L that channels the misfolded protein to the dislocon where proteins are retrotranslocated to the cytosol (45). Though the dislocon is not well-characterized it is thought to include Sec61, the E3 ligase HRD1, Derlins 1-3, and ATPase VCP/p97, which converts the energy released by ATP hydrolysis into mechanical force for the extraction process (4, 45). BiP and PDIs (ERdj5 and ERp90), also play a role, at this stage, binding and oxidizing the proteins to be transported (41, 59). In the cytosol, ubiquitin activating enzymes (E1), conjugating enzymes (E2), and E3 ligases (primarly HRD1 and AMFR) transfer ubiquitin to the protein in an ATP-dependent process (46). Finally, ubiquitinated proteins are degraded by the 26S proteasome in a pathway that also consumes ATP (45, 46). Overall the ERAD is an extremely costly process that consumes ATP during the extraction, ubiquitination and degradation of misfolded proteins (4, 41, 45) (Table 1 and Figure 2).

Secretory proteins that achieve the correct folded and assembled conformation are then transported from the ER to the Golgi complex in coat protein complex II (COPII) carrier vesicles (47). Vesicles for anterograde (forward) transport are formed in ER exit sites (ERES) and key proteins involved in their assembly include SEC12, SEC16, secretion-associated Ras-related 1 (SAR1) GTPase and the two major coat subunits SEC23- SEC24 and SEC13- SEC31 (47, 60). Proteins enter the vesicles by diffusion (bulk-flow), or undergo selective uptake (cargo capture) by SEC24, and other receptors (2, 48, 61). Upon leaving the ERES COPII vesicles shed their SEC13- SEC31 coats in a process that involves the hydrolysis of GTP bound to SAR1 (62), and fuse with one another or with the ER-Golgi intermediate compartments (ERGIC) (2, 47). Rab-family small GTPases and Rab effectors are involved in vesicle tethering to the membranes. After tethering vesicles fuse with the acceptor compartment delivering the cargo to the Golgi (2, 48). Retrieval of misplaced ER luminal proteins from the ERGIC or from the Golgi is achieved by retrograde transport in coat protein complex I (COPI) carrier vesicles. Formation of COPI vesicles involves the ADP-ribosylation factor 1 (ARF) GTPase, that recruits the coatomer subunits to the membrane (48). Vesicle formation, coat shedding and tethering to target membranes are energy consuming processes that consume GTP (2). SAR1 and ARF GTPases switch between inactive GDP-bound and active GTP-bound forms. Guanine nucleotide exchange factors (GEFs) catalyze the release of GDP (allowing the union of a new GTP molecule); while GTPase-activating proteins (GAPs) promote the hydrolysis of bound GTP, regulating enzyme activity and therefore vesicle trafficking (2, 63) (Table 1).

From the *cis*-Golgi compartment proteins are transported via cisternal maturation to the trans-Golgi network (48), where proteins to be secreted are sorted in secretory vesicles and delivered to the plasma membrane, to the endosomal system or to immature secretory granules (33, 48). Motor proteins, kinesin, and dynein transport the cargo vesicles along microtubules. Kinesins transport secretory vesicles from the organelles where they arise to the cortical region, while dynein mediates their transport in the opposite direction (49, 64). Myosin V and II are responsible for the movement of vesicles along actin filaments in the cortical area (49, 50). The cortical actin network, at the cell borders, acts as a diffusion barrier that prevents the access of granules to secretory sites until the arrival of the triggering stimuli (50). Motor proteins, kinesin, dynein and myosin use the energy released during ATP hydrolysis to move vesicles along microtubules and actin filaments (9) (Table 1 and Figure 2).

Upon arrival to the plasma membrane secretory proteins are released to the extracellular space by exocytosis. Constitutive exocytosis occurs in all cell types to release extracellular proteins and maintain plasma membrane homeostasis and cell polarity. While regulated exocytosis occurs in specialized secretory cells and is triggered by secretion signals (13), such as increases in Ca^2+^ concentrations (65). Calcium ions bind sensor molecules and promote the fusion of vesicles with the plasma membrane (65). Upon the arrival of the secretion stimuli myosin II drives the movement of vesicles to the fusion sites where they dock to the plasma membrane (66). SNARE complexes (formed by syntaxin, SNP-25, and VAMP), and SM proteins (such as Munc18-1), are in charge of the fusion of vesicles with the plasma membrane (10, 13, 51), while the actin and myosin network contributes to the release of vesicle contents in an ATP-dependent manner (50, 66, 67). Fusion of vesicles with target membranes also consumes ATP. SNARE proteins localized in vesicles and target membranes interact forming a complex that pulls the two membranes together, exerting the force required for fusion. The dissociation of the SNARE complex involves soluble NSF attachment protein (SNAP) and the ATPase N-ethylmaleimide-sensitive fusion protein (NSF) that hydrolyses ATP (10, 51) (Table 1 and Figure 2).

Overall the concerted action of many cellular components is required to ensure the selective and efficient secretion of proteins. Many of these steps require energy and mitochondria play a relevant role, fulfilling the ATP requirements of the pathway (Figures 1, 2). Besides, mitochondria help to modulate the intracellular concentrations of Ca^2+^ (13, 14), an essential cofactor in the folding process and a potent trigger of exocytosis (65) (Figure 1).

### Unconventional Pathways for Protein Secretion

Though the conventional protein secretion pathway was considered for a long time as the only mechanism for protein secretion, we now know that many proteins use alternative pathways. These include the secretion of cytosolic proteins that do not have a signal peptide (leaderless proteins) or a transmembrane domain; and proteins that contain a signal peptide or a transmembrane domain and enter the ER but are not transferred to the Golgi complex (68).

Proteins without a leader sequence are secreted along three different pathways: Type I pathways involve the translocation from the cytoplasm to the extracellular space through a pore in the plasma membrane. In Type II secretion processes the ATP-binding cassette transporter is responsible for the secretion of the protein present in the cytoplasm while in Type III secretion the proteins use autophagosomes and endosomes to reach the extracellular space. The Golgi-bypassing route is also known as Type IV secretion pathway (68). Some proteins are constitutively secreted through unconventional pathways, however these pathways are usually induced in stressful conditions, such as nutrient starvation, inflammation, and mechanical or ER stress (32, 68).

One of the most studied proteins secreted by the unconventional pathways is interleukin-1β (IL-1β) (68, 69). During inflammation IL-1β is produced as an immature and inactive form that is cleaved by caspase 1 upon recruitment and activation by the Nod-like receptor family pyrin domain containing 3 (NLRP3) inflammasome (70), in a process that will be discussed below in detail. The assembly and activation of the complex is an energetically costly event that requires the hydrolysis of ATP by NLRP3 (71). The posterior secretion of mature IL-1β involves the formation of a pore that increases the permeability of the plasma membrane (69) or occurs through the autophagosome/endosmal pathway (68).

### Secretion Pathway of Neurotransmitters

Many neurotransmitter molecules are amino acids or amino acid derivatives and their secretion occurs through exocytosis. The pathway starts with the synthesis of the neurotransmitters by enzymes, followed by their loading into of synaptic vesicles. The loading process involves transporters in the vesicle membrane and happens at the expense of an electrochemical proton gradient, generated by the vacuolar H^+^-ATPase. This pump uses the energy released by ATP hydrolysis to transport protons into the vesicle lumen (72). Loaded vesicles can then be sequestered in a reserve pool or travel to specialized release sites, known as active zones, at the presynaptic terminal where vesicles become docked at the plasma membrane. Increases in Ca^2+^ concentrations promote the fusion of the vesicle with the plasma membrane and discharge their content into the synaptic cleft (73). Calcium sensing, vesicle docking and exocytosis follow the same steps described for the conventional secretion pathway of proteins (51). These steps are followed by the recovery of the synaptic vesicle membranes that are recycled for refilling in preparation for the next cycle of exocytosis (74).

## Overview of Mitochondrial Structure, Function and Dynamics

### Mitochondrial Bioenergetics

Mitochondria are cell organelles defined by a double membrane, the outer mitochondrial membrane (OMM) and the inner mitochondrial membrane (IMM). The OMM separates the mitochondria from the cytosol, however the voltage-dependent anion channel (VDAC or porin) allows the passage of metabolites and ions. In the OMM are also located proteins involved in apoptosis, mitochondrial dynamics, and tethering to other organelles. In the IMM, two functional and structurally different regions are described: the inner boundary membrane and the cristae, where electron transport and ATP synthesis take place in a process known as oxidative phosphorylation (75). In the cristae we find the electron transport chain (ETC) complexes: NADH-ubiquinone oxidoreductase (complex I), succinate-ubiquinone oxidoreductase (complex II), ubiquinol-cytochrome c oxidoreductase (complex III), and cytochrome c oxidase (complex IV), as well as the F_o_F_1_-ATP synthase (ATP synthase or complex V) and two mobile electron carriers, ubiquinone and cytochrome c (75).

The ETC complexes are assembled forming supercomplexes that optimize electron transport and proton shuttling through the IMM (76, 77). ATP synthase localizes on the edges of cristae forming dimers, which optimize ATP production (78). At the cristae junctions we find the mitochondrial contact site and cristae-organizing system (MICOS), a large oligomeric complex that interacts with both the IMM and OMM (79). Cristae structure influences the ETC, complex and supercomplex formation, and can present different morphologies depending on the metabolic state of the cell (80).

Mitochondria are the main source of ATP in most cells, since many catabolic pathways converge in this organelle and result in the production of ATP by oxidative phosphorylation. The catabolism of metabolites such as glucose, proteins, and fatty acids produces acetyl-CoA, which is in turn oxidized in the tricarboxylic acid cycle generating the reduced electron donors nicotinamide adenine dinucleotide (NADH) and flavin adenine dinucleotide (FADH_2_). Electrons are then transferred to the ETC and flow through the complexes to molecular oxygen (O_2_). This thermodynamically favorable electron transport releases energy, which is used to pump protons from the matrix to the intermembrane space, at complexes I, III, and IV, creating an electrochemical gradient (81). The energy released by the dissipation of the electrochemical proton gradient is then coupled to the synthesis ATP from ADP and Pi, catalyzed by ATP synthase (82, 83).

### Reactive Oxygen Species in Mitochondria

Mitochondrial catabolism of nutrients and electron transport in the respiratory chain involves many redox reactions that have as by-products reactive oxygen species (ROS) (84). In particular, superoxide anion radical is formed by several flavoproteins and at complex I and III of the respiratory chain, and its dismutation gives rise to hydrogen peroxide (85). These mildly oxidant species can result in the formation of higher oxidant species such as peroxynitrite, formed in the reaction between superoxide and nitric oxide, oxo-metal complexes, nitrogen dioxide, and hydroxyl radical (86).

Under physiological conditions mitochondria produce controlled levels of oxidants, many of which participate in signaling processes. However, ROS formation can increase during cellular stress or in pathological conditions. Although mitochondrial oxidants can be detoxified by enzymatic and non-enzymatic antioxidants (87, 88), an imbalance in mitochondrial redox status may lead to mitochondrial damage (84, 89, 90). In fact, oxidants impair the activity of enzymes of the tricarboxylic acid cycle, the ETC, and ATP synthase (84, 90).

### Mitochondrial Dynamics

Mitochondrial dynamics consists of fusion and fission events driven mainly by dynamin-related GTPases (91). Optic atrophy protein 1 (OPA1) and mitofusins 1 and 2 (MFN1 and MFN2) are involved in mitochondrial fusion. The long OPA1 isoform is anchored to the inner membrane, where it promotes IMM fusion. The soluble and short OPA1 isoform, found in the intermembrane space, maintains mitochondrial cristae structure (92). MFN1 and MFN2 participate in tethering and fusion of the outer membranes of two different mitochondria through the formation of homo and heteroligomeric complexes (93, 94). Phospatidic acid, a fusogenic lipid formed in a reaction catalyzed by mitochondrial phospholipase D, is also required for OMM fusion (95).

In turn, mitochondrial fission is carried out by the GTPase dynamin related protein 1 (DRP1), a cytosolic protein that is recruited to the OMM (96) by receptor proteins, such as mitochondria fission factor (97), mitochondrial dynamics protein of 49 and 51 kDa (98) and fission 1 protein (FIS1) (99). DRP1 oligomerizes around mitochondria into a ring-like structure constricting the organelle (96). ER-mitochondrial contacts play a role in mitochondrial fission, determining the position of the fission events (100) and ER tubules wrap around mitochondria and constrict them (100). Components of the cytoskeleton such as actin, myosin II and septin 2 are required for mitochondrial fission (101–104), as well as the ER protein inverted formin 2 (105) and a mitochondrial Spire1 isoform (103). The latter two cooperate to induce localized actin polymerization at the constriction sites (103). Both fusion and fission are highly regulated processes, and changes in protein levels, GTPase activity and post-translational modifications of proteins involved in mitochondrial dynamics affect not only mitochondrial morphology but also cellular bioenergetics and homeostasis (106).

Multiple studies linking mitochondrial morphology and bioenergetics can be found in the literature (107–109). At the molecular level several reports support that MFN2 is required for correct mitochondrial function (110); since its silencing results in an impairment of oxidative phosphorylation, while overexpression enhances mitochondrial metabolism (19, 111–113). Less evidence supports a role for MFN1. Although there are reports on MFN1 silencing decreasing mitochondrial respiration (114) this does not seem to be the case in all models (19). OPA1 deletion also affects mitochondrial membrane potential, respiration and cristae structure and reduces assembly, and stability of supercomplexes (80, 114–117).

In order to face increases in energy demands, such as those imposed by secretion, mitochondria also modify their cellular distribution. Motor proteins, kinesin, and dynein, transport mitochondria over long distances along microtubules toward the cell periphery or the cell center, respectively (49). While myosin 19, mediates short-range movement along the actin filaments near the cell periphery (49, 118). The mitochondrial Rho GTPase (MIRO) and the adaptor proteins trafficking kinesin-binding proteins 1 and 2 (TRAK1 and 2) support bidirectional mitochondrial movement along microtubules by binding kinesin and dynein (119–121) (Figure 2). MIRO proteins also engage myosin 19, linking mitochondria to actin filaments (122) and interact with components of the MICOS complex, linking the transport machinery to cristae organization and ensuring the appropriate provision of energy to the regions where mitochondria are delivered (123).

### ER-Mitochondria Contact Sites (ERMCs)

Mitochondria can interact with several cellular compartments, including organelles involved in protein secretion such as the Golgi complex and ER (124–126). In particular, mitochondrial interactions with the ER have been extensively studied and many components and functions of the ER–mitochondrial contact sites (ERMCs)^1^ have been identified. ERMCs are crucial sites for the synthesis and exchange of lipids, Ca^2+^ transport, mitochondrial fission and inflammasome formation (4, 125, 127).

Among the proteins found in ERMCs is MFN2, and though some controversy exists (128, 129), this protein is proposed to link the ER membrane to mitochondria through interactions with MFN1 and MFN2 in the OMM (130, 131). The mitochondrial proteins MITOL and mitostatin, as well as the ER protein presenilin 2 modulate these MFN2-dependent interactions between organelles (132–134). Other relevant proteins linking mitochondria to the ER are VDAC, the OMM chaperone GRP-75 and the inositol-1,4,5-trisphosphate-sensitive channel or receptor (IP3R) in the ER. Another tethering complex is formed between the ER protein vesicle-associated membrane protein- associated protein B (VAPB) and the OMM protein tyrosine phosphatase-interacting protein 51(PTPIP51) (135–137) (Figure 2).

Physical tethering of mitochondria and the ER by mitofusins (130, 131), VDAC/IP3R/GRP-75 (14, 138), and VAPB/ PTPIP51 (135) is required for efficient transport of Ca^2+^ between organelles. Besides, several regulatory proteins modulate Ca^2+^ release from the ER and uptake by mitochondria, through interactions with IP3R or VDAC (123, 138, 139). Of particular interest is the chaperone calnexin, whose translocation to different ER domains is regulated by palmitoylation of two conserved cysteine residues (140) and interaction with regulatory proteins (138). Calnexin migration to the ERMCs is required for Ca^2+^ transfer from the ER to the mitochondria (141).

Calcium ion leaves the ER through the I3PR and enters the mitochondria through VDAC and the mitochondrial calcium uniporter (MCU) complex present in the OMM and IMM, respectively (14, 138). The complex is formed by the MCU, a pore that allows the passage of the ions through the lipid membrane (142, 143), essential MCU regulator (ERMES) and mitochondrial calcium uptake 1 and 2 proteins (MICU1 and 2) that act as gatekeepers regulating Ca^2+^ uptake (144). Calcium entry to mitochondria occurs at the expense of the H^+^ gradient in the IMM (144). In the matrix, Ca^2+^ activates several dehydrogenases (including enzymes of the tricarboxylic acid cycle) and ATP synthase enhancing oxygen consumption rate and ATP synthesis (15, 144). Thus, MERCs are extremely important for mitochondrial function. On the other hand Ca^2+^ uptake by mitochondria prevents or “buffers” the increase of Ca^2+^ concentrations near the mouth of the IP3R affecting its activity as we will describe in detail in the next section (14).

In ERMCs we can also find SAR1 and ARF. These small GTPases involved in COPII and COPI vesicle assembly are also required to maintain mitochondrial morphology and function (145, 146). SAR1 regulates the size of mitochondrial ER contact sites by affecting the curvature of the membrane (146). While ARF1 and its guanine nucleotide exchange factor (GBF1) interact with mitochondrial protein MIRO, and their depletion affects mitochondrial morphology, autophagy, positioning, and movement within the cell (145, 147). These observations point to an important role of secretory pathway components in dynamics and physiology of mitochondria.

Overall, mitochondria are dynamic organelles whose function, morphology, distribution, and contacts with other organelles (49, 109, 148) undergo changes in response to intracellular energy requirements, as well as other physiological and pathological stimuli (109).

## Mitochondria in Secretion

### Fulfilling ATP Requirements of the Secretion Process

As mentioned before, many of the steps in the conventional secretion pathway demand energy (Table 1 and Figures 1, 2). Chaperones involved in protein translocation to the ER and protein folding consume ATP (5–7), as does protein quality control by the ERAD (8), transport and fusion of vesicles with target membranes (2, 9, 51). Calcium signaling during regulated exocytosis also consumes ATP to maintain the levels of IP3 precursors and to pump calcium from the cytoplasm into the ER or to the extracellular space (13, 14, 149). Overall, an increase in secretion of proteins is bound to challenge the cell's bioenergetics capacity; and inhibition of ATP synthesis, using inhibitors or uncouplers of oxidative phosphorylation, prevents the secretion of many proteins (7, 150–152).

The mechanism for ATP transport into the ER in mammalian cells was elusive for many years, and different possibilities, ranging from the existence of specific transporters to non-specific transport through anion channels or leaky membranes, have been proposed (4). However, recently protein SLC35B1 was identified as an ATP/ADP exchanger present in the ER membrane, and named AXER (153). Silencing the expression of SLC35B1/AXER reduced ATP levels in the ER of different cell lines and affected BiP dependent protein import activity and Ca^2+^ levels inside the ER (153, 154). A decrease in ATP in the ER was observed upon inhibition of SERCA or exposure to high cytosolic Ca^2+^ concentrations, suggesting that a Ca^2+^ gradient across the ER membrane is required for ATP transport into the ER (154). Inhibitors and uncouplers of oxidative phosphorylation lead to a decrease in ATP levels inside the ER as well, suggesting mitochondria is a relevant source of ATP for the ER (154, 155). Interestingly, several components of the mitochondrial ATP synthase complex immunoprecipitate with SLC35B1/AXER; along with proteins involved in ER protein import and folding such as BiP, calnexin, and Sec61 complex (153). Transfer of ATP from mitochondria to the ER probably occurs at ERMCs, where both organelles are in close proximity, yet this is still to be established (4).

### Calcium Buffering by Mitochondria in Exocytosis

As we mentioned above, Ca^2+^ signals for exocytosis in many secretory cells. The increase in cytosolic Ca^2+^ concentration triggers vesicle fusion with the plasma membrane leading to the release of the cargo to the extracellular space. Calcium enters the cytosol through channels in the plasma or the ER membranes and several processes can affect the magnitude and duration of the signal for exocytosis, among them is Ca^2+^ uptake by mitochondria (13). Mitochondria are located in close proximity to Ca^2+^ channels in the ER and plasma membrane and can take up the ion through the VDAC and MCU channels in the OMM and IMM, respectively (144). This process is known as Ca^2+^ buffering and is driven by the negative potential across the IMM (144). Positioning of mitochondria near the sites of Ca^2+^ entry to the cytosol is also a requirement for the efficient uptake of the ion and effective Ca^2+^ signaling for exocytosis. This depends on proteins involved in mitochondrial dynamics and movement, such as MFN2, myosin, kinesin, TRAK, and MIRO (14, 156–158). Of note, MIRO is both an adaptor protein, that links mitochondria to TRAK and kinesin, and a Ca^2+^ sensor which can regulate mitochondrial trafficking in response to changes in Ca^2+^ concentrations (157).

Ca^2+^ buffering has different effects on exocytosis, depending on the channel and cell type. Voltage-gated Ca^2+^ channels (Ca_v_) are present in the plasma membrane. These are activated by membrane depolarization and mediate Ca^2+^ influx in response to action potentials and other depolarizing signals (159). The increase in cytosolic Ca^2+^ stimulates exocytosis by binding syaptogamins that promote SNARE mediated vesicle fusion with the membrane (65). Mitochondria can be found in the cell cortex regions, near the sites of exocytosis and in this setting Ca^2+^ sequestering by mitochondria reduces vesicle release (157, 160, 161).

In the plasma membrane we also find store-operated channels, known as Ca^2+^ release-activated **(**CRAC) channels. These are composed by Orai pore proteins, present in the plasma membrane, and STIM proteins from the ER and are activated by depletion of calcium stores in the ER (13, 149, 158). When Ca^2+^ levels fall in the ER STIM proteins migrate to ER-plasma membrane junctions where they activate Orai channels, allowing the entry of Ca^2+^ into the cytosol. In turn, high cytosolic Ca^2+^ concentrations result in the closure of the pore and both a rapid and a slow Ca^2+^-dependent inactivation have been described (158). Mitochondrial translocation to the plasma membrane is essential to sustain Ca^2+^ influx through CRAC channels (162), since it prevents the slow inactivation of the CRAC channel without affecting the fast Ca^2+^-dependent inactivation (158). Inhibitors and uncouplers of electron transport and oxidative phosphorylation that dissipate the proton gradient prevent Ca^2+^ uptake by mitochondria and promote channel inactivation (163). Inhibition of kinesin- dependent transport of mitochondria to the plasma membrane also results in channel inactivation (162).

In the ER membrane the IP3R and ryanodine-sensitive receptors are the channels responsible for Ca^2+^ release to the cytosol (13, 14). IP3R is activated by IP3, a second messenger formed by hydrolysis of phosphatidylinositol 2,5-biphosphate by phospholipase C. While ryanodine- sensitive receptors in secretory processes are activated by Ca^2+^ to release Ca^2+^ stored in the ER, in a process known as Ca^2+^ induced Ca^2+^ release that amplifies Ca^2+^ signaling (149). For both channels the response to cytosolic Ca^2+^ concentrations is bell shaped, since Ca^2+^ can act both as a positive or negative regulator of the channel depending on the concentration of the ion (14, 164). Thus, high concentrations of cytosolic Ca^2+^ result in a decrease in the activity of both I3PR and ryanodine sensitive receptors (14, 164). Uptake of Ca^2+^ by mitochondria prevents its accumulation precluding channel inhibition by high concentrations of Ca^2+^ (14).

From what we just described it is clear that mitochondria contribute to the regulation of intracellular Ca^2+^ concentrations, and therefore impact on the regulated exocytosis of many proteins and other signaling molecules such as neurotransmitters (Figure 1).

### Mitochondria in the Activation of NLRP3 Inflammasome

The NLRP3 inflammasome is a multiprotein complex that participates in innate immunity. It is activated by multiple signals of infection, cellular damage, or stress, to produce inflammatory cytokines that trigger innate immune responses (71, 165). The sensor molecule NLRP3, the adaptor protein ASC and the effector caspase-1 form the complex, and recently the NIMA-related kinase 7 was identified as a fourth component. Upon stimulation, NLRP3 oligomerizes and recruits ASC that in turn recruits caspase 1. The assembly of the NLRP3 inflammasome leads to the activation of caspase 1 that then cleaves pro- IL-1β and pro-IL-18, producing the mature cytokines IL-1β and IL-18 (71). As mentioned above the assembly of the NLRP3 inflammasome requires energy that is obtained from ATP hydrolysis catalyzed by NLRP3 NACHT domain (71). Mitochondria supplies ATP for this process but contributes also in other ways to the activation of the inflammasome.

NLRP3 inflammasome activation occurs in two steps, priming and NLRP3 activation. Priming involves the recognition of pathogen-associated molecular patterns (PAMPs, such as LPS) by pattern recognition receptors or binding of cytokines to receptors. These events lead to the activation of NF-κB and consequent upregulation of NLRP3, caspase 1 and IL-1β and IL-18 gene expression (71). The second step is the activation of NLRP3 by PAMPs or damage-associated molecular patterns (DAMPs, such as ATP); which involves multiple signaling events that result in the assembly of the complex [for exhaustive reviews see (71, 165)]. Mitochondrial ROS and oxidized mtDNA released into the cytosol can trigger NLRP3 activation (16, 17).

Moreover, mitochondria are required for the assembly of the inflammasome. In absence of stimuli the NLRP3 is found in the cytoplasm associated with the ER, while its activation results in an association with mitochondria and enrichment in ERMCs (17). ASC and caspase 1 also migrate to mitochondria upon activation and are enriched at ERMCs (17, 18). In a recent report Elliot et al. suggest that priming results in NLRP3 and capase 1 linkage to the OMM, by association with the phospholipid cardiolipin, and mtROS are proposed to mediate cardiolipin transfer from the IMM to the OMM. Instead the association of ASC with mitochondria occurs in the activation step and does not require cardiolipin, but is dependent on NLRP3 and regulated by cytosolic Ca^2+^ (18). Interestingly in absence of VDAC, a relevant OMM channel and tether molecule in mitochondria-ER contact sites, activation is not observed (17).

Other mitochondrial proteins that are required for NLRP3 inflammasome activation are mitochondrial antiviral signaling proteins (MAVS). These proteins form aggregates in the OMM, associate with NLRP3 and promote its oligomerization, and activation of the inflammasome during RNA virus infections (166). The fusion protein MFN2 also participates in inflammasome activation and its silencing reduces IL-1β secretion in response to viral infection (167). Finally, dynein dependent transport of mitochondria along microtubules, toward the ER, is required for the activation of the inflammasome (168). Acetylation and deacetylation of tubulin by acetyltransferase MEC17 and the NAD^+^ dependent deacetylase sirtuin 2 regulate microtubule transport of mitochondria to the ER and inflammasome activation (168).

In sum, mitochondria provide energy, signaling molecules and a structural scaffold for the assembly and activation of the NLRP3 inflammasome (Figure 1).

## Bioenergetic Failure and Secretory Defects in Disease

Considering the relevant roles of mitochondria in secretion it is not surprising that mitochondrial dysfunction underlies the pathogenesis of certain diseases. Herein we discuss the role of this organelle in some relevant physiological secretion processes, and present evidence linking bioenergetic failure to the development of disease.

### Insulin Secretion by Pancreatic β-Cells in Diabetes Mellitus

Pancreatic β-cells are secretory cells located in the islets of Langerhans in the pancreas. Their main function is to synthesize and secrete insulin, a peptidic hormone responsible for regulating levels of glucose in the blood. Upon the increase in glucose concentrations in plasma, β-cells secrete insulin stored in the secretory granules and increase the synthesis of the hormone (169, 170).

Mitochondria play a key role in glucose-stimulated insulin secretion (GSIS) in pancreatic β-cells. In these cells glucose is metabolized in the glycolytic pathway and tricarboxylic acid cycle and results in ATP synthesis by oxidative phosphorylation. ATP promotes the closure of ATP-sensitive potassium channels and depolarization of the plasma membrane, leading to the opening of voltage-gated Ca^2+^ channels. Calcium influx then triggers the exocytosis of insulin granules (170). This process is termed K_ATP_- dependent GSIS. A K_ATP_- independent GSIS that depends on mitochondrial GTP and phosphoenolpyruvate metabolism has also been described (171, 172). Therefore, GSIS is extremely sensitive to genetic interventions and pharmacological agents that affect mitochondrial metabolism (22). This is clearly exemplified by the work of Kennedy et al. showing that depletion of mitochondria in the INS-1 insulin-secreting cell line precludes insulin secretion upon exposure to glucose (173). Likewise impairment of autophagy in pancreatic β-cells affects both mitochondrial respiration and GSIS, affecting glucose metabolism *in vivo* (174). As does inhibition of Ca^2+^ uptake by mitochondria after silencing of MCU or MICU1 gene expression, that also reduces ATP synthesis and insulin secretion (175).

Pancreatic β-cells present an active mitochondrial network where mitochondria constantly undergo fusion and fission events (176); and functional mitochondrial dynamics are required for GSIS in INS-1 cells and primary β-cells (23). Overexpression of FIS1 in human pancreatic β-cells resulted in fragmentation and clustering of the mitochondrial network in the perinuclear area, a decrease in mitochondrial membrane potential and ATP synthesis, along with impairment of GSIS (177). Similar results where observed upon silencing of FIS1 expression that affected respiration rates and insulin secretion (178), overexpression of a dominant negative form of fission protein DRP1 and DRP1 silencing (178, 179). Alteration in the fusion machinery also affected insulin secretion. Overexpression of MFN1 resulted in hyperfusion and aggregation of mitochondria, reduced ATP levels and GSIS (177); while OPA1 ablation impaired glucose-stimulated oxygen consumption rate, ATP production and insulin secretion (116).

It is clear that mitochondria play relevant roles in pancreatic β-cell secretion and a growing body of evidence links mitochondrial dysfunction to impaired insulin release in diabetes mellitus. Diabetes mellitus are a group of metabolic disorders characterized by hyperglycemia, resulting from defects in insulin secretion, insulin sensitivity, or both (180). In type 1 diabetes the cause is a total deficiency of insulin secretion, while in type 2 diabetes the cause is a combination of resistance to insulin action of the liver, skeletal muscle, and adipose tissue and an inadequate compensatory insulin secretory response (180, 181).

To start with, diabetes mellitus (type 1 and 2) is frequently observed in patients with inherited mitochondrial diseases, which are caused by defects in mtDNA or in nuclear genes encoding mitochondrial proteins that affect mitochondrial ATP synthesis (27). Several point mutations as well as deletions and rearrangements in mtDNA are strongly associated with the onset of diabetes (27, 182). As are mutations in nuclear genes encoding proteins involved in maintenance of the mitochondrial genome (27). The most common defect is the mutation m.3243A>G in MT-TL1, which encodes a mitochondrial tRNA for leucine (183) and can lead to maternally inherited mitochondrial diabetes and deafness (MIDD) (27). Other point mutations in mitochondrial tRNA genes MT-TL1, MT-TK, MT-TS2, MT-TE, and MT-TT as well as in MT-ND6, a core subunit of complex I, have also been associated with diabetes mellitus (27, 184). Patients, carrying the m.3243A>G mutation presented reduced pancreatic insulin secretion (185–187), yet increases in insulin resistance and alterations in glucose metabolism cannot be discarded as causes of the disease (188).

Additionally profound changes in mitochondrial metabolism have been observed in pancreatic islets of type 2 diabetes patients and animal models. These include down regulation of components of the tricarboxylic cycle, ETC, ATP synthase and proteins involved in transport across the OMM and IMM (29, 189); as well as a decrease in glucose oxidation and a loss of glucose-stimulated increases in NADH, ATP, and oxygen consumption (29, 190–192). Other authors have observed an upregulation of uncoupling protein 2 (UCP-2), an IMM protein that dissipates the proton gradient; in pancreatic islets in type 2 diabetes (190, 193, 194). Genetic deletion of UCP-2 in mice enhances islet ATP generation and insulin secretion during glucose stimulation and reduces hyperglycemia in the ob/ob mice (a well-known diabetes type 2 mouse model), providing further support for the role of UCP-2 in the pathogenesis of the disease (194). Regardless of the mechanism, the impairment in mitochondrial function in pancreatic β cells precludes the glucose-induced increase in ATP levels. Lack of ATP will prevent the closure of ATP-sensitive K^+^ channels (that is required to activate insulin secretion), impair the steps of the secretion pathway that require energy, resulting in lower levels of the circulating hormone and hyperglycemia.

### Neurotransmitter Release in Alzheimer's Disease

Neurons are excitable cells that communicate with other cells at synapses. At the chemical synapse neurotransmitters are released by pre-synaptic neurons and bind their receptor at the post-synaptic terminal of target cells. Vesicle discharge at the synaptic cleft occurs in response to an influx of Ca^2+^ ions through voltage-gated Ca^2+^ channels, triggered by the action potential. Three different pools of synaptic vesicles can be found in neurons readily releasable pool, the recycling pool, and the reserve pool. The vesicles in the readily releasable pool are released under moderate or intense neuronal activity, while vesicles from the reserve pool are recruited only upon intense stimulation. The latter constitute the majority of vesicles in presynaptic terminals (28, 160).

Maintaining the electrochemical gradients required for action potentials, transporting, discharging, recycling, and refilling synaptic vesicles, and regulating Ca^2+^ concentrations are energy demanding processes, that require functional mitochondria (28, 195). Regarding secretion in particular, loading of vesicles with neurotransmitters requires ATP to fuel the vacuole type H^+^-ATPase (72). Besides, the mobilization of the reserve pool requires mitochondrial ATP that is used by myosins to move the vesicles along actin filaments (196). Additionally, Ca^2+^ signaling for vesicle exocytosis requires a constant export of the ion from the cytosol to the extracellular space, the ER and mitochondria. Plasma membrane Ca^2+^ ATPase (PMCA) and SERCA at the ER actively consume ATP (149). Mitochondria provide ATP for these pumps and directly remove Ca^2+^ from the cytosol through the MCU, as described previously (28, 196). Therefore, mitochondria are very abundant in neurons, where they form a highly dynamic network and their position and morphology change continually (28). In particular we find a high number of mitochondria at chemical synapses, where they provide ATP and buffer local rises in Ca^2+^ concentrations (156, 160, 197). Since mitochondrial biogenesis and elimination (by mitophagy) occurs mainly in the neuronal soma, mitochondria travel between the axon terminals and the cell body (156) via the microtubule network. Motor proteins kinesin (anterograde) and dynein (retrograde), adaptor proteins TRAK, MIRO, and syntabulin as well as fission protein DRP1 are involved in mitochondrial trafficking (28, 156, 196). Docking to microtubules, by syntaphilin, or to actin filaments limits mitochondrial mobility, retaining these organelles near to the sites where energy is required (156, 160).

Due to their high-energy demands, neurons are extremely affected in mitochondrial diseases, caused by mutations in mtDNA, and in other pathologies where mitochondrial function is compromised (30, 198). An example of the latter is Alzheimer's disease (AD), a neurodegenerative disease that involves progressive synaptic dysfunction and death of neurons in cortical and subcortical regions. AD is characterized by the accumulation of extracellular plaques of the peptide amyloid-β (Aβ) and the formation of intracellular tangles of the microtubule-associated protein tau (198, 199).

Alterations in energy metabolism can be found in patients and animal models of AD. Reduced glucose metabolism was observed in brain regions affected by AD and the reduction correlated with cognitive decline in AD patients (200, 201). Analysis of brain samples from patients with AD showed abnormalities in mitochondrial cristae of hippocampal neurons (202); and reduced activity of enzymes involved in oxidative metabolism, namely pyruvate dehydrogenase (PDH), α-ketoglutarate dehydrogenase and complex IV (203–205). Studies of brain mitochondrial function in a transgenic AD mouse model also revealed decreases in PDH, complex IV and respiratory control ratios (RCR) and the decay in PDH and neuronal mitochondrial function preceded plaque formation (206). Moreover, in recent reports mitochondrial dysfunction was detected in hippocampal synaptosomes from transgenic AD rat model (207, 208). Decreased complex I activity, oxygen consumption rate, and ATP synthesis, along with an increase in hydrogen peroxide formation were measured, evidencing bioenergetics defects as well as oxidative stress at the presynaptic level (208). Alterations in mitochondrial dynamics and motility have also been observed in transgenic animal models of AD, and result in lower number of mitochondria at the presynapses (28, 209, 210). Regarding the causes behind bioenergetic failure in neurons, the Aβ peptide has been found to accumulate in mitochondria (206, 211), where it directly impairs complex IV activity and ATP synthesis, and increases ROS formation (212). Aβ can also directly affect mitochondrial dynamics, promoting mitochondrial fragmentation (213, 214). Defects in Ca^2+^ handling by mitochondria have also been observed in AD and Aβ can alter Ca^2+^ homeostasis at several levels (28, 73). Hyperphosphorylated tau also affects mitochodrial respiration and ATP synthesis, through the inhibition of complex I, impairs mitochondrial trafficking and induces mitochondrial fission (215). Overall in AD patients impaired mitochondrial energy metabolism, dynamics, Ca^2+^ homeostasis and oxidant formation are affected in neurons, at presynaptic terminals where neurotransmitter secretion takes place (28, 198).

Synapse loss is an early event in AD animal models and patients, and exhibits a strong correlation with cognitive deficits (73). Presynaptic release of neurotransmissors is affected in AD, and Aβ can affect various steps of the secretion process including SNARE mediated fusion, recovery of vesicle membranes by endocytosis and Ca^2+^ homeostasis (73). The decrese in mitochondrial ATP synthesis and Ca^2+^ buffering, observed in AD at the presynaptic level, probably affects neurotransmitter release as well, and should be considered in the design of pharmacological strategies to target the disease (215). Supporting this concept are results obtained in a transgenic AD rat model, where treatment with a pharmacological inducer of mitochondrial biogenesis helped to recover mitochondrial function and protected against cognitive impairment (208).

## Mitochondria and Secretion in Other Pathological Settings

In this section we present two examples of mitochondrial involvement in secretion processes that underlie pathology. This is not an extensive list, but rather a couple of interesting examples that support the idea that mitochondria might be interesting targets in the design of drugs to modulate secretory processes.

### Mast Cells Secretion in Atopic Dermatitis

Mast cells are cells of hematopoietic origin that participate in host defense and immunity as well as tissue repair, wound healing, angiogenesis and are also responsible for the development of allergies (216). Mast cells can be activated by foreign pathogens, toxins chemical agents and particles, as well as by mediators of the immune response, such as immunoglobulin E (IgE) and complement components (216, 217). Upon activation mast cells release pre-stored mediators, and/or *de novo* synthesize and secrete a wide range of biologically active molecules (216). Pre-stored mediators include proteases, histamine, proteoglycans, and cytokines such as tumor necrosis factor (TNF), these are released immediately upon activation and are critical for initiating mast cell-mediated innate immune responses. Differently from pre-stored mediators, other molecules are synthesized *de novo* (i.e., lipids, cytokines, chemokines, antimicrobial peptides, growth and angiogenic factors) and play multiple roles in the immune response and other physiological and pathological processes (216, 217).

Mast cell degranulation and TNF secretion requires Ca^2+^ and mitochondrial ATP (31, 218). Interestingly stimuli that induce mast cell degranulation, promote the translocation of mitochondria to sites of exocytosis, close to the cell surface; and pharmacological or genetic inhibition of DRP1 inhibits both TNF secretion and mitochondrial translocation, in a cell line derived from human mast cell leukemia (24). In this report authors also show that calcium is required for DRP1 activation, as well as for mitochondrial translocation, and postulate that calcineurin is involved in DRP1 recruitment to mitochondrial surface (24). The relevance of this observation was further supported by studies in human mast cells from patients with atopic dermatitis where increased degranulation and mitochondrial translocation was also observed, along with an increase in DRP1 and calcineurin expression (24). Mast cell degranulation also results in the secretion of mitochondrial particles outside cells including mtDNA, that in turn promote cytokine release from other mast cells, keratinocytes, and endothelial cells promoting inflammation (219).

### Senescence Associated Secretory Phenotype in Disease

Cellular senescence is triggered in response to stress stimuli. Agents that damage DNA and strong mitogenic signals, such as the expression of oncogenes or loss of tumor suppressors, are strong senescence inducers (220–223). Senescent cells are characterized by permanent proliferation arrest, activation of the DNA damage response (DDR), increase in size and β-galactosidase activity and acquisition of a secretory phenotype (224, 225). The senescence-associated secretory phenotype (SASP) includes numerous cytokines and chemokines (e.g., IL-1α, IL-1β and IL-6, IL-8), growth factors and extracellular proteases. These mediators can induce senescence in neighboring cells, alter the extracellular matrix, contribute to local inflammation, and recruit immune cells among other effects (226–228); and contribute to several physiological and pathological processes (229, 230). At the transcriptional level most of the SASP components are regulated by NF-κB, C/EBPβ, and NOTCH (231). IL-1β is a component of the SASP that is secreted through unconventional pathways (32). However, many of the SASP factors are most probably secreted through the conventional pathway, though this is practically unexplored.

Several studies support that establishment of senescence and the secretory phenotype is accompanied by metabolic reprogramming including increases in mitochondrial oxygen consumption rates, biogenesis and dynamics. In particular, important alterations have been observed in oncogene and therapy induced senescence (19–21, 232–235). Interestingly replicative senescent cells that present mitochondrial dysfunction (236, 237) secrete lower levels of cytokines and other mediators than oncogene induced senescent cells (20, 224).

Moreover, mitochondria are required to sustain the SASP and mitochondrial depletion down regulates the expression and secretion of multiple cytokines and other factors in senescent cells (21, 26). In oncogene induced senescence inhibition of carnitine palmitoyl transferase I (CPTI), a key enzyme of the mitochondrial fatty acid oxidation pathway, decreases the secretion of several cytokines and growth factors, but not of IL-1β (20). Similar results were obtained upon inhibition of nicotinamide phosphoribosyltransferase (NAMPT), and enzyme from the NAD^+^ synthesis pathway (25), or silencing of mitochondrial sirtuin 3, known to impair mitochondrial electron transport (26, 238). Silencing of mitochondrial fusion proteins, MFN1 and 2 also impaired the secretion of IL-6 in senescent cells (19). Since mitofusins participate in the tethering of the mitochondria to the ER, these results suggest the association between these two organelles might be relevant in secretion processes in senescence. Finally mitochondrial ROS formation by the ETC (239, 240) and NADPH oxidase 4 (241, 242) are increased in senescent cells, and contribute to the establishment of senescence and the SASP [through the activation of NF-kB (243) and the DDR (244–246)]. Taken together these results point to a link between mitochondrial oxidative metabolism and dynamics and the proinflammatory secretory phenotype.

Since the SASP has been implied in numerous diseases (229, 247), the search for senolytics (that will kill senescent cells) or senomorphics (that will modulate the SASP) has become an active area of research (248, 249). Mitochondria appear as promising targets for the modulation of the SASP in pathological settings were senescent cells are involved.

## Concluding Remarks

Herein we present evidence that mitochondria are required for the successful export of proteins to the extracellular space. Mitochondrial dynamics, bioenergetics and distribution as well as their interactions with other organelles, in particular with the ER, can undergo profound changes in response to secretion stimuli. Mitochondria contribute and support the secretion of proteins providing ATP for energy requiring processes, buffering Ca^2+^ concentrations and offering structural support and signals for NLRP3 inflammasome activation. A better understanding, at the molecular level, of the role of mitochondria in secretion processes is required and will help in the development of new genetic and pharmacological strategies to modulate protein secretion in pathological contexts.

## Author Contributions

JM, IM, DT, and CQ contributed to the writing of the manuscript, CQ supervised all the activities. JM made the figures. All authors reviewed contents and approved the final version of the manuscript.

## Conflict of Interest

The authors declare that the research was conducted in the absence of any commercial or financial relationships that could be construed as a potential conflict of interest.

## Footnotes

^1^ERMCs are also known as MERCs or mitochondria-associated ER membranes (MAMs).

## Abbreviations

ARF: ADP-ribosylation factor
BiP: ER chaperone BiP
Ca^2+^: calcium
COPI: coat protein complex I
COPII: coat protein complex II
DRP1: GTPase dynamin related protein 1
EDEM: ER degradation-enhancing alpha-mannosidase-like proteins
ER: endoplasmic reticulum
ERAD: endoplasmic reticulum- associated degradation
ERES: ER exit sites
ERGIC: ER-Golgi intermediate compartment
ERMCs: ER–mitochondrial contact sites
ETC: electron transport chain
FIS1: fission 1 protein
GRP: glucose regulated protein
GSIS: glucose-stimulated insulin secretion
GT: UDP-glucose:glycoprotein glucosyltransferase
IL-1β: interleukin-1β
IMM: inner mitochondrial membrane
IP3: inositol-1, 4, 5-trisphosphate
IP3R: inositol-1, 4, 5-trisphosphate- sensitive channel or receptor
MCU: mitochondrial calcium uniporter
MICOS: mitochondrial contact site and cristae-organizing system
MICU: MCU regulator and mitochondrial calcium uptake
MFN1: mitofusin 1
MFN2: mitofusin 2
MIRO: mitochondrial Rho GTPase
NLRP3: Nod-like receptor family, pyrin domain containing 3
OMM: outer mitochondrial membrane
OPA1: optic atrophy protein 1
ROS: reactive oxygen species
SAR1: secretion associated Ras related 1 GTPAse
SASP: senescence-associated secretory phenotype
SERCA: sarcoplasmic/endoplasmic reticulum calcium ATPase
SRP: signal recognition particle
TNF: tumor necrosis factor, VDAC, voltage-dependent anion channel.
